# Supplementary material for: Flavescence Dorée Phytoplasma Has Multiple ftsH Genes that Are Differentially Expressed in Plants and Insects
Source: Int J Mol Sci. 2019 Dec 24;21(1):150. doi: 10.3390/ijms21010150 (PMC6981957; doi:10.3390/ijms21010150)
Supplement: Supplementary file 1 [file ijms-21-00150-s001.pptx]

## Slide 1
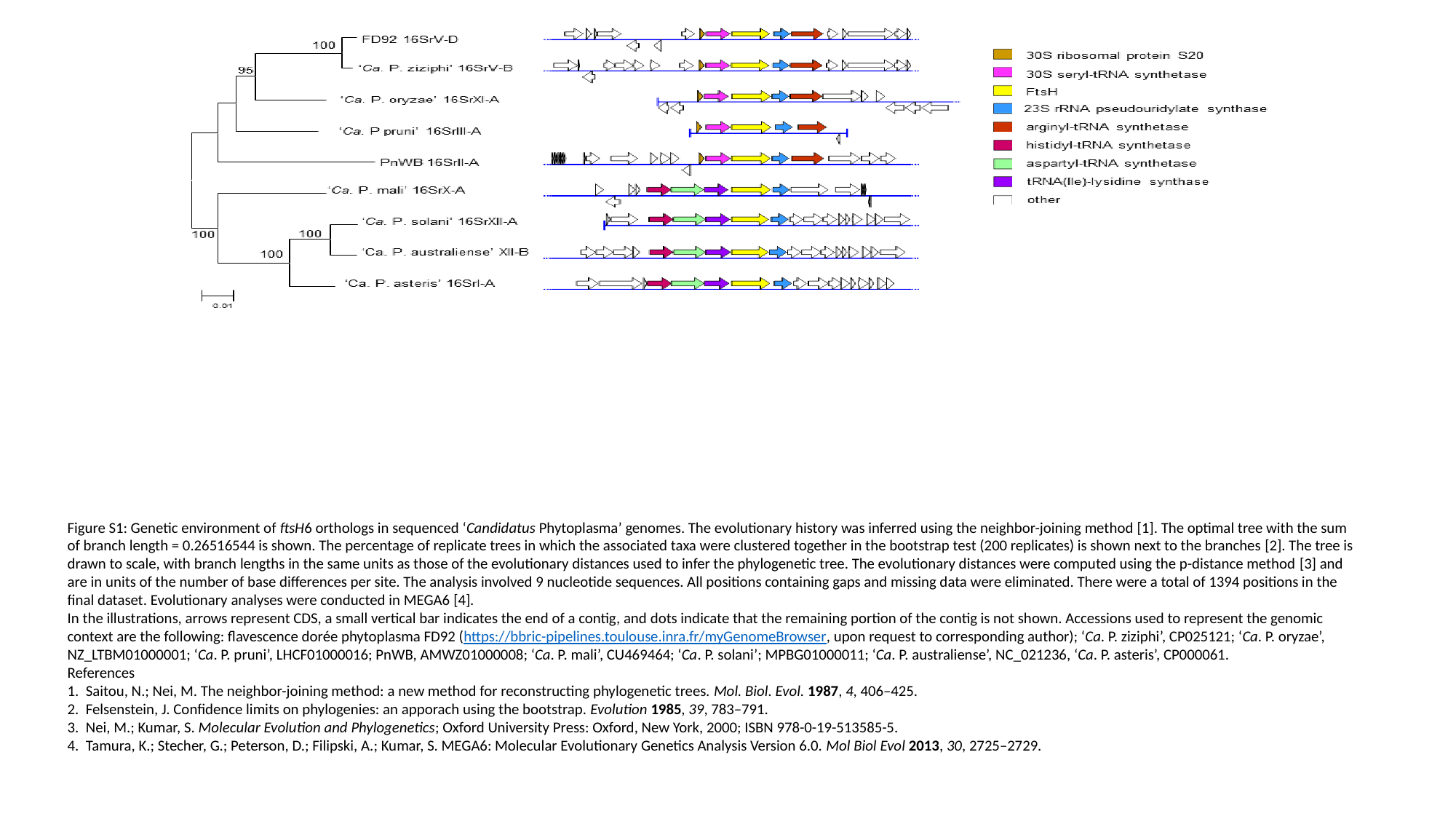

Figure S1: Genetic environment of ftsH6 orthologs in sequenced ‘Candidatus Phytoplasma’ genomes. The evolutionary history was inferred using the neighbor-joining method [1]. The optimal tree with the sum of branch length = 0.26516544 is shown. The percentage of replicate trees in which the associated taxa were clustered together in the bootstrap test (200 replicates) is shown next to the branches [2]. The tree is drawn to scale, with branch lengths in the same units as those of the evolutionary distances used to infer the phylogenetic tree. The evolutionary distances were computed using the p-distance method [3] and are in units of the number of base differences per site. The analysis involved 9 nucleotide sequences. All positions containing gaps and missing data were eliminated. There were a total of 1394 positions in the final dataset. Evolutionary analyses were conducted in MEGA6 [4].
In the illustrations, arrows represent CDS, a small vertical bar indicates the end of a contig, and dots indicate that the remaining portion of the contig is not shown. Accessions used to represent the genomic context are the following: flavescence dorée phytoplasma FD92 (https://bbric-pipelines.toulouse.inra.fr/myGenomeBrowser, upon request to corresponding author); ‘Ca. P. ziziphi’, CP025121; ‘Ca. P. oryzae’, NZ_LTBM01000001; ‘Ca. P. pruni’, LHCF01000016; PnWB, AMWZ01000008; ‘Ca. P. mali’, CU469464; ‘Ca. P. solani’; MPBG01000011; ‘Ca. P. australiense’, NC_021236, ‘Ca. P. asteris’, CP000061.
References
1. Saitou, N.; Nei, M. The neighbor-joining method: a new method for reconstructing phylogenetic trees. Mol. Biol. Evol. 1987, 4, 406–425.
2. Felsenstein, J. Confidence limits on phylogenies: an apporach using the bootstrap. Evolution 1985, 39, 783–791.
3. Nei, M.; Kumar, S. Molecular Evolution and Phylogenetics; Oxford University Press: Oxford, New York, 2000; ISBN 978-0-19-513585-5.
4. Tamura, K.; Stecher, G.; Peterson, D.; Filipski, A.; Kumar, S. MEGA6: Molecular Evolutionary Genetics Analysis Version 6.0. Mol Biol Evol 2013, 30, 2725–2729.
